# Effects of peach branch organic fertilizer on the soil microbial community in peach orachards

**DOI:** 10.3389/fmicb.2023.1223420

**Published:** 2023-07-07

**Authors:** Chenyu Liu, Defeng Han, Haiqing Yang, Zhiling Liu, Chengda Gao, Yueping Liu

**Affiliations:** ^1^College of Bioscience and Resources Environment, Beijing University of Agriculture, Beijing, China; ^2^Pinggu District of Fruit Bureau, Beijing, China; ^3^College of Humanities and Urban-Rural Development, Beijing University of Agriculture, Beijing, China; ^4^Key Laboratory for Northern Urban Agriculture Ministry of Agriculture and Rural Affairs, Beijing University of Agriculture, Beijing, China

**Keywords:** peach branch organic fertilizer, peach yield, peach quality, soil chemical property, soil bacterial community

## Abstract

Peach branches is a by-product of peach industry. Making peach branch waste into peach branch organic fertilizer (PBOF) is a promising strategy of ecological utilization. In this study, the effects of PBOF on the yield and quality of peach fruit, chemical properties of bulk soil, and soil bacterial communities were investigated in a peach orchard. The results showed that the yield and sugar/acid ratio of two high-level PBOF treatments (SDH.4 and SKR.4) was higher than no fertilization treatment (CK), but there was no significant difference compared to the commercial organic fertilizer treatment (SYT.4). Moreover, the three fertilizer treatments increased soil nutrients such as soil organic matter (SOM) and available potassium (AK), compared to CK. Furthermore, PBOF increased the relative abundance of beneficial bacteria, and enhanced the soil bacterial co-occurrence pattern and the potential function of bacterial communities to degrade exogenous compounds. In addition, thanks to the local policy of encouraging the use of PBOF, the use cost of PBOF is lower than commercial organic fertilizer, which is conducive to the development of ecological agriculture.

## 1. Introduction

Peach [*Prunus persica* (L.) Batsch] is a fruit tree within the family Rosaceae. Peach fruit is rich in a variety of nutrients beneficial to the human body and it has become one of the most popular fruits due to its sweet and delicious taste and beautiful attractive appearance. The origin of the peach is China, which has a history spanning thousands of years (Liu et al., 2015). At present, China’s peach production ranks first in the world; it has increased from approximately 4.6 Mt. to approximately 16 Mt., an increase of approximately 248% in the past 20 years. China’s peach area harvested has increased from approximately 0.45 Mh to approximately 0.83 Mh, an increase of approximately 84% (FAO, 2022). With the rapid development of the peach industry and the demand for rural ecological environment governance, how to deal with a large amount of peach branch waste generated by pruning peach trees has become a difficult problem. Burning peach branch waste or stacking it in the field will cause greenhouse gas emissions, which can seriously endanger ecological balance and human health. Previous studies showed that peach branch waste can be used as raw material to extract and prepare a reinforcing agent and as a promising feedstock for ethanol production (Buratti et al., 2018; Wei et al., 2023). In agriculture, peach branch waste can be used as a substrate for oyster mushrooms (Yang et al., 2022). However, there is less research on the return of peach branch waste to the field. At present, peach branch waste is crushed mechanically and composted by adding livestock and poultry manure, biological agents, and straw decomposition agents to make peach organic fertilizer in many peach-producing areas in China. This process has formed a recycling mode in which peach branch waste is transformed into renewable resources, and then renewable resources are transformed into agricultural resources again. The conversion of discarded peach branches into organic fertilizer will significantly alleviate environmental pollution. Moreover, with the widespread utilization of peach branch organic fertilizer (PBOF), the local farmers will enjoy substantial economic benefits from the sale of these peach branch wastes.

Fertilization plays a vital role in enhancing agricultural production, serving as a crucial method to not only improve soil quality but also increase soil productivity (Yang et al., 2019). The increase in crop yield in recent decades has depended largely on chemical fertilizer (Geng et al., 2019; Iqbal et al., 2021); for example, suitable potassium fertilizer can often improve fruit quality (Crisosto and Costa, 2008). However, excessive and long-term application of chemical fertilizers has negative effects on ecosystems, including soil and water (Sun et al., 2015; Pirttilä et al., 2021). Excessive fertilization can lead to soil acidification and a decrease in soil organic matter (SOM) content, which directly affects crop growth and sustainable use of soil. Among them, SOM plays a critical role in maintaining soil fertility and productivity (Lin et al., 2019; Wu et al., 2020). To increase SOM content, improve soil quality, and enhance soil fertility, applying organic fertilizer is an excellent choice (Liang et al., 2014; Hua et al., 2020). Organic fertilizer treatment has been shown to significantly affect soil chemical properties, increasing SOM, total nitrogen (TN), and available phosphorus (AP; Ji et al., 2021). Moreover, the application of organic fertilizer alleviates soil acidification by adjusting the pH of the soil (Afreh et al., 2018). In the study of Ye J. H. et al. (2022), similar results were found, and the application of organic fertilizer stabilized the soil pH within the optimal range. Furthermore, the application of organic fertilizer has a good effect on yield and quality by improving soil nutrients, properties, and microecology (Liu et al., 2021).

Soil microorganisms are very active in the soil ecosystem, are involved in the decomposition of organic matter and nitrogen fixation, and play a crucial role in soil energy flow and the nutrient cycle (Morris and Blackwood, 2015; Nazaries et al., 2021; Wang T. et al., 2022). The soil bacterial community affects processes such as carbon cycling, litter decomposition, and soil fertility changes (Cao et al., 2021; Zhang H. H. et al., 2022). Fertilization has an impact on soil microbial abundance and community structure (Guo et al., 2020; Iqbal et al., 2022). Research by Wang et al. (2016b) showed that the application of bioorganic fertilizer had a significant effect on the structure and composition of the soil microbial community in apple orchards, such as increasing the relative abundance of the genera *Bacillus*, *Lysobacter*, and *Pseudomonas*. The difference is that the application of chemical fertilizer did not increase the relative abundance of *Bacillus*, *Lysobacter*, and *Pseudomonas.* There was a correlation between the abundance of soil microorganisms and yield; for example, the relative abundance of *Bacillus* had a positive correlation with yield. Similar results were obtained in another study (Wang et al., 2016a). In addition, the application of organic fertilizer increased nutrient availability and enhanced synergistic interactions between soil microorganisms, thereby promoting plant growth (Zhang et al., 2019). Li et al. (2021) found the different responses of bacterial communities to plant-derived and animal-derived organic fertilizer. The animal-derived organic fertilizer significantly increased the Richness index and Shannon index, while plant-derived organic fertilizer did not. Compared with animal-derived organic fertilizer, plant-derived organic fertilizer had a weaker influence on the abundance of each phylum level. Liu et al. (2020) showed that the Shannon index and Chao1 index did not increase with increasing organic fertilization levels. The relative abundance of Actinobacteria, Verrucomicrobia, and Armatimonadetes decreased with increasing organic fertilization levels. Moreover, the relative abundance of *Pseudomonas* increased with increasing organic fertilization levels. Overall, the influence of fertilizer categories and levels on soil microorganisms cannot be ignored.

Peach branch waste composting is an effective mode to improve the resource utilization of peach orchard waste. However, the effects of PBOF on soil properties and bacterial community, peach fruit quality and orchard yield and their relationship with each other have not been systematically studied. The main objectives of this study were to (1) study the effects of PBOF on soil properties and the soil bacterial community; (2) study the effects of PBOF on the quality and yield of peach fruit; (3) explore the potential correlation between soil properties and the soil bacterial community and peach fruit quality and yield; and (4) compare the effects of PBOF and commercial organic fertilizer. There were two main hypotheses: (1) peach fruit quality and yield are affected by the soil bacterial community and soil properties and (2) PBOF can achieve similar results to the application effect of commercial organic fertilizer. This study aimed to provide a theoretical basis for scientific fertilization, ensure the yield and quality of peach fruit, and reduce the environmental pollution caused by peach branch waste.

## 2. Materials and methods

### 2.1. Experimental location and experimental design

This study began in 2019 in Houbeigong Village, Dahuashan Town, Pinggu District, Beijing (40°15′N, 117°3′E). Pinggu is the main producing area of peaches in Beijing. The region has a monsoon climate at medium latitudes, with a large temperature difference between day and night, sufficient sunshine, annual precipitation of approximately 630 mm, and an average annual temperature of 11.7°C. The soil is classified as Haplustalf (United States Department of Agriculture). The chemical properties of the peach orchard soil were showed in Supplementary Table S1. The variety of peach planted in the peach orchard is “Wan no. 24”, and the planting distance between peach trees is 2 m × 4 m (480 plants ha^−1^).

The experimental study was built on a randomized complete block design with a total of 10 treatments (each with 3 replicates): no fertilization, 0 kg/667 m^2^ (CK); application of Dahua PBOF, 2560 kg/667 m^2^ (SDH.2); application of Dahua PBOF, 3840 kg/667 m^2^ (SDH.3); application of Dahua PBOF, 5120 kg/667 m^2^ (SDH.4); application of Kerui PBOF, 2560 kg/667 m^2^ (SKR.2); application of Kerui PBOF, 3840 kg/667 m^2^ (SKR.3); application of Kerui PBOF, 5120 kg/667 m^2^ (SKR.4); application of Yite commercial organic fertilizer, 2,560 kg/667 m^2^ (SYT.2); application of Yite commercial organic fertilizer, 3,840 kg/667 m^2^ (SYT.3); and application of Yite commercial organic fertilizer, 5,120 kg/667 m^2^ (SYT.4). Dahua PBOF, Kerui PBOF and Yite commercial organic fertilizer were purchased from Beijing Dahua Fertilizer Industry Co., Ltd., Beijing Kerui Compound Fertilizer Co., Ltd., and Beijing Yite Organic Fertilizer Factory, respectively. At present, there are few studies on the application effect of PBOF, and only two kinds of PBOF are produced in the region: Dahua and Kerui. The chemical properties of the three fertilizers were showed in Supplementary Table S1. All other practices were carried out in accordance with normal field management methods.

### 2.2. Determination of peach yield and quality

Five peach fruits were randomly selected from different locations on three peach trees. A total of 15 fruits were picked per treatment. Soluble solids content (SSC) and titratable acid (TA) were determined using a handheld near infrared spectrometer (NIRMagic3100, Beijing Weichuang Yingtu Technology Co., Ltd., China). The sugar/acid ratio (SAR) was the ratio of SSC and TA. A balance was used to measure the weight of a single fruit and calculate the yield based on the number of fruits.

### 2.3. Soil sampling

We collected bulk soil samples in October 2019. Three peach trees were selected with the same treatment. Two points approximately 1 m from the south and north sides of the trunk were selected for soil sampling at a depth of 20–40 cm. The samples from both points were mixed, the roots and stones were removed from the mixed samples through a sieve (2 mm), and the samples were divided into two parts. One part was air-dried, which was used to determine the soil chemical properties. The other part was stored at-80°C for DNA extraction. The soil sampling method of this study was modified according to the method by Wang et al. (2016b).

### 2.4. Analysis of soil chemical properties

The following chemical properties were examined: pH, soil organic matter (SOM), alkali-hydrolysable nitrogen (AN), available phosphorus (AP), available potassium (AK), Ca, Cu, Fe, Mn, Mg, and Zn. Soil pH was determined with soil to water ratio of 1:10 using a digital pH meter (FE28, Mettler-Toledo) (Du et al., 2021). For proper determination of soil chemical properties, samples were collected after well air-dried until reaching a constant weight. SOM was determined by the potassium dichromate volumetric method (Xiao et al., 2022). For AN, we used the alkali hydrolysis diffusion method (Gu et al., 2021). AP was determined by the molybdenum-antimony-scandium-based colorimetry method after extraction by sodium bicarbonate (Du et al., 2022). AK was extracted with ammonium acetate and determined by flame photometry (Shen et al., 2008). Ca, Cu, Fe, Mn, Mg, and Zn were determined by inductively coupled plasma–optical emission spectrometry (ICP–OES, iCAP6300, Thermo Scientific, United States).

### 2.5. DNA extraction, PCR amplification, and illumina sequencing

Genomic DNA was extracted from 0.5 g soil samples using the DNeasy PowerSoil Kit according to the manufacturer’s instructions (QIAGEN, Germany). DNA quality and quantity were verified with 1% agarose gel and NanoDrop 2000 (Thermo Scientific, United States). The V3-V4 variable regions of the bacterial 16S rRNA gene were amplified using primers 343F (TACGGRAGGCAGCAG) and 798R (AGGGTATCTAATCCT) with barcodes (Nossa et al., 2010). Two rounds of PCR amplification were carried out. The PCR mixture of the first round (30 μL) consisted of 15 μL 2 × Gflex PCR buffer, 1 μL forward and reverse primers, 0.6 μL Tks Gflex DNA Polymerase, 50 ng DNA template, and ddH_2_O. The thermal cycling conditions of the first round were as follows: initial denaturation at 94°C for 5 min, 26 cycles of 94°C for 30 s, 56°C for 30 s, and 72°C for 20 s, and a final extension at 72°C for 5 min. The PCR mixture of the second round (30 μL) consisted of 15 μL 2 × Gflex PCR buffer, 1 μL Adapter I5 and Adapter I7, 0.6 μL Tks Gflex DNA Polymerase, 50 ng first round PCR product, and ddH_2_O. The thermal cycling conditions of the second round were as follows: initial denaturation at 94°C for 5 min, 7 cycles of 94°C for 30 s, 56°C for 30 s, and 72°C for 20 s, and a final extension at 72°C for 5 min. The final products were purified with VAHTS DNA Clean Beads (Vazyme, China) and quantified using a Qubit dsDNA Assay Kit (Life Technologies, United States). Sequencing was performed by Shanghai OE Biotech Co., Ltd., (China) on an Illumina NovaSeq PE250 platform (Illumina, United States).

### 2.6. Bioinformatics analysis and data processing

The raw data were analyzed using Trimmomatic software (version 0.35) for quality control (Bolger et al., 2014). The paired-end reads were assembled using Flash software (version 1.2.11; Magoč and Salzberg, 2011). Using VSEARCH software (version 2.4.2), the reads were clustered into operational taxonomic units (OTUs) with 97% similarity (Rognes et al., 2016). The representative read of each OTU was selected, and the representative reads were classified according to the Silva database (version 132) using RDP classifiers, with a confidence threshold of 70% (Wang et al., 2007). Bioinformatic analysis was performed on the Tutools platform,^1^ OECloud tools,^2^ and Wekemo Bioincloud.^3^ Venn diagrams, bacterial community analysis, correlation analysis, network analysis, and PICRUSt2 were calculated and drawn in R. The linear discriminant analysis (LDA) effect size (LEfSe) was realized on the website http://huttenhower.sph.harvard.edu/galaxy/, and biomarkers of soil bacteria in each treatment were identified, and all bacterial taxa had LDA scores >2 (Xu et al., 2022). Microsoft Excel 2021 was used to calculate peach yield and quality for different treatments. Statistical analysis was conducted using OriginPro 2022b (OriginLab Corp., United States). Using Fisher’s LSD test (*p* < 0.05) to compare the significance, one-way analysis of variance (ANOVA) was used to analyze the differences in the yield and quality of peaches, soil chemical properties, α-diversity index (Chao1 index and Shannon index), soil bacterial community, and functional prediction between different treatments.

## 3. Results

### 3.1. Peach yield and quality and soil chemical properties

All fertilization treatments increased yield and SAR, and as the levels of fertilization increased, yield and SAR also increased accordingly. The SAR in SDH.4 and SKR.4 treatments were significantly (*p* < 0.05) higher compared to CK, but there is no significant difference between the three types of fertilizers or the different levels of fertilization. About yield, the SDH.4, SKR.4, and SYT.4 treatments increased yield by 21.46%, 23.51%, and 27.82%, respectively, compared to CK. The high-level fertilization (SDH.4, SKR.4, and SYT.4) showed a significantly (*p* < 0.05) higher production compared to the low-level fertilization (SDH.2, SKR.2, and SYT.2). However, there is no significant difference between the three types of fertilizers in yield (Table 1).

**Table 1:** Effects of different fertilization treatments on peach fruit yield and quality.

Treatment	Yield (kg/667m^2^)	SAR
CK	3178.07 ± 528.896c	34.38 ± 2.711b
SDH.2	3327.07 ± 27.916c	37.03 ± 3.950ab
SDH.3	3592.28 ± 220.051bc	38.52 ± 0.402ab
SDH.4	3860.00 ± 130.400ab	40.07 ± 5.306ab
SKR.2	3372.50 ± 72.317c	37.30 ± 1.140ab
SKR.3	3499.52 ± 104.960bc	38.42 ± 3.673ab
SKR.4	3925.15 ± 204.192ab	41.49 ± 6.012a
SYT.2	3302.21 ± 495.168c	37.44 ± 2.812ab
SYT.3	3577.39 ± 94.471bc	38.39 ± 1.927ab
SYT.4	4062.14 ± 199.232a	41.58 ± 6.562a

SAR, sugar/acid ratio; CK, no fertilization, 0 kg/667 m^2^; SDH.2, Dahua PBOF, 2560 kg/667 m^2^; SDH.3, Dahua PBOF, 3840 kg/667 m^2^; SDH.4, Dahua PBOF, 5120 kg/667 m^2^; SKR.2, Kerui PBOF, 2560 kg/667 m^2^; SKR.3, Kerui PBOF, 3840 kg/667 m^2^; SKR.4, Kerui PBOF, 5120 kg/667 m^2^; SYT.2, Yite commercial organic fertilizer, 2,560 kg/667 m^2^; SYT.3, Yite commercial organic fertilizer, 3,840 kg/667 m^2^; SYT.4, Yite commercial organic fertilizer, 5,120 kg/667 m^2^.The different letters in the table indicate a significant difference at *p* < 0.05.

Compared to CK, all fertilization treatments increased AN, AK, AP, and SOM, while with a weak impact on pH. There were no significant differences in AN, AP, and SOM between the three types of fertilizers or the different levels of fertilization. The SKR.3 and SKR.4 treatments increased AN by 30.77% and 38.46%, respectively, compared to CK, while the SKR.4, SYT.3, and SYT.4 treatments increased AP by 46.67%, 53.33%, and 60.00%, respectively. Additionally, the SDH.2, SDH.3, SDH.4, SKR.4, and SYT.4 treatments increased SOM by 40.78%, 40.63%, 40.83%, 49.07%, and 44.62%, respectively, relative to CK. The medium-level fertilization (SYT.3) and high-level fertilization (SYT.4) showed a significantly (*p* < 0.05) higher AK compared to the low-level fertilization (SYT.2). Moreover, the AK of the SDH.4, SKR.2, SYT.3 and SYT.4 treatments were significantly (*p* < 0.05) higher than that of CK. The pH of SYT.3 treatments was significantly (*p* < 0.05) higher than that of the SKR.3 treatment (Table 2).

**Table 2:** Effects of different fertilization treatments on soil chemical properties.

Treatment	AN (mg/kg)	AK (mg/kg)	AP (mg/kg)	SOM (g/kg)	pH
CK	126.70 ± 10.006b	399.07 ± 50.305d	145.98 ± 14.806c	19.79 ± 2.061c	7.37 ± 0.339ab
SDH.2	167.00 ± 22.068ab	614.24 ± 110.761bcd	160.20 ± 13.865bc	27.86 ± 6.949ab	7.21 ± 0.110ab
SDH.3	164.21 ± 18.803ab	649.88 ± 134.228bcd	189.71 ± 16.990abc	27.83 ± 3.870ab	7.20 ± 0.221ab
SDH.4	170.36 ± 9.641ab	794.22 ± 213.390bc	174.82 ± 3.146abc	27.87 ± 1.353ab	7.04 ± 0.152b
SKR.2	168.75 ± 19.524ab	731.11 ± 153.914bc	173.98 ± 2.919abc	26.27 ± 2.188abc	7.07 ± 0.173ab
SKR.3	174.96 ± 53.873a	634.34 ± 46.173bcd	189.85 ± 28.077abc	23.26 ± 4.041abc	6.96 ± 0.101b
SKR.4	182.68 ± 14.533a	656.22 ± 22.177bcd	222.91 ± 92.265ab	29.50 ± 2.003a	7.14 ± 0.190ab
SYT.2	148.09 ± 25.510ab	566.77 ± 101.258 cd	174.01 ± 22.474abc	21.01 ± 4.380bc	7.08 ± 0.586ab
SYT.3	163.61 ± 19.075ab	1087.46 ± 205.957a	230.38 ± 20.139a	23.65 ± 1.645abc	7.45 ± 0.188a
SYT.4	171.85 ± 48.770ab	870.88 ± 301.069ab	241.88 ± 76.922a	28.62 ± 9.434ab	7.40 ± 0.148ab

AN, alkali-hydrolysable nitrogen; AK, available potassium; AP, available phosphorus; SOM, soil organic matter. The definitions of CK, SDH.2, SDH.3, SDH.4, SKR.2, SKR.3, SKR.4, SYT.2, SYT.3, and SYT.4 are shown in Table 1. The different letters in the table indicate a significant difference at *p* < 0.05.

### 3.2. Soil bacterial community diversity

**Figure 1:**
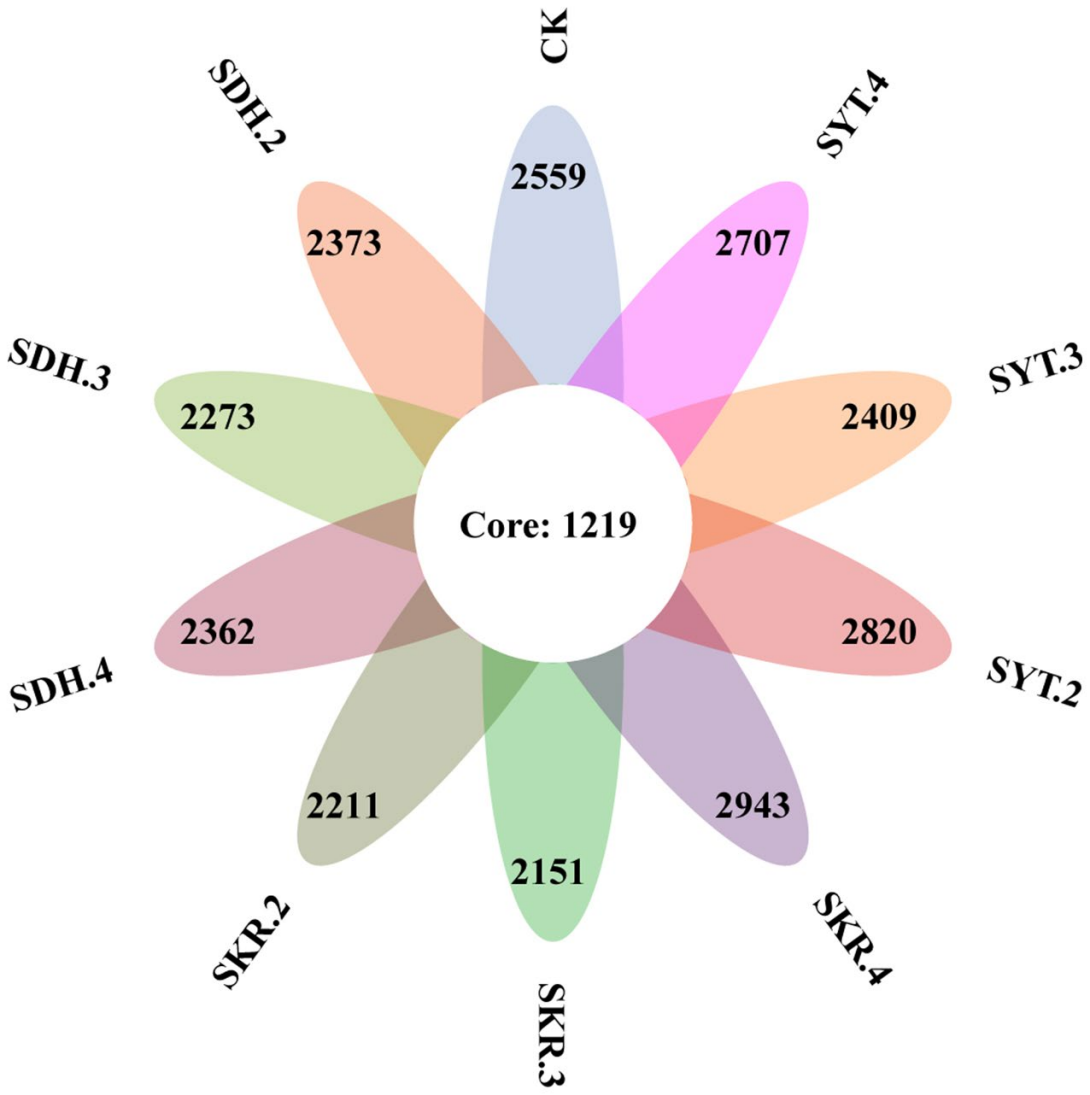
Venn diagram showing the common and unique bacterial OTUs under different fertilizer treatments. CK, no fertilization, 0 kg/667 m^2^; SDH.2, Dahua PBOF, 2560 kg/667 m^2^; SDH.3, Dahua PBOF, 3840 kg/667 m^2^; SDH.4, Dahua PBOF, 5120 kg/667 m^2^; SKR.2, Kerui PBOF, 2560 kg/667 m^2^; SKR.3, Kerui PBOF, 3840 kg/667 m^2^; SKR.4, Kerui PBOF, 5120 kg/667 m^2^; SYT.2, Yite commercial organic fertilizer, 2,560 kg/667 m^2^; SYT.3, Yite commercial organic fertilizer, 3,840 kg/667 m^2^; SYT.4, Yite commercial organic fertilizer, 5,120 kg/667 m^2^.

Based on 97% sequence similarity, a total of 30 phyla, 87 classes, 202 orders, 334 families, 713 genera, and 7,359 bacterial OTUs were identified. The number of common OTUs in the 10 treatments were 1,219, and the number of unique OTUs in the CK, SDH.2, SDH.3, SDH.4, SKR.2, SKR.3, SKR.4, SYT.2, SYT.3, and SYT.4 treatments were 2,559, 2,373, 2,273, 2,362, 2,211, 2,151, 2,943, 2,820, 2,409, and 2,707, respectively (Figure 1). The SKR.4 treatment had the highest number of unique OTUs.

Table 3 shows the variations in the Chao1, and Shannon indices under different fertilization treatments. There was no significant difference in the Chao1 index between all fertilization treatments. The Shannon index of the high-level fertilization (SKR.4) was significantly (*p* < 0.05) higher than that of the medium-level fertilization (SKR.3) and low-level fertilization (SKR.2). Compared to that of CK, the Shannon index of the SKR.4 and SYT.4 treatments increased. Compared to that of CK, the Chao1 index of the SKR.4 treatment and the Shannon index of the SKR.4 and SYT.4 treatments increased. Among all treatments, the Chao1 and Shannon indices of the SKR.4 treatment were the highest.

**Table 3:** Effects of different fertilization treatments on the alpha diversity index.

Treatment	Chao1 index	Shannon index
CK	3574.35 ± 276.787a	6.67 ± 0.911ab
SDH.2	3386.85 ± 327.351a	6.20 ± 0.642b
SDH.3	3231.31 ± 416.232a	6.22 ± 0.901b
SDH.4	3376.26 ± 287.881a	6.19 ± 0.688b
SKR.2	3233.20 ± 455.258a	6.11 ± 0.894b
SKR.3	3184.11 ± 320.215a	5.92 ± 0.424b
SKR.4	3809.40 ± 284.195a	7.71 ± 1.063a
SYT.2	3489.00 ± 720.015a	6.53 ± 1.371ab
SYT.3	3417.72 ± 292.480a	6.27 ± 0.535b
SYT.4	3466.94 ± 411.396a	6.82 ± 0.529ab

The definitions of CK, SDH.2, SDH.3, SDH.4, SKR.2, SKR.3, SKR.4, SYT.2, SYT.3, and SYT.4 are shown in Table 1. The different letters in the table indicate a significant difference at *p* < 0.05.

**Figure 2:**
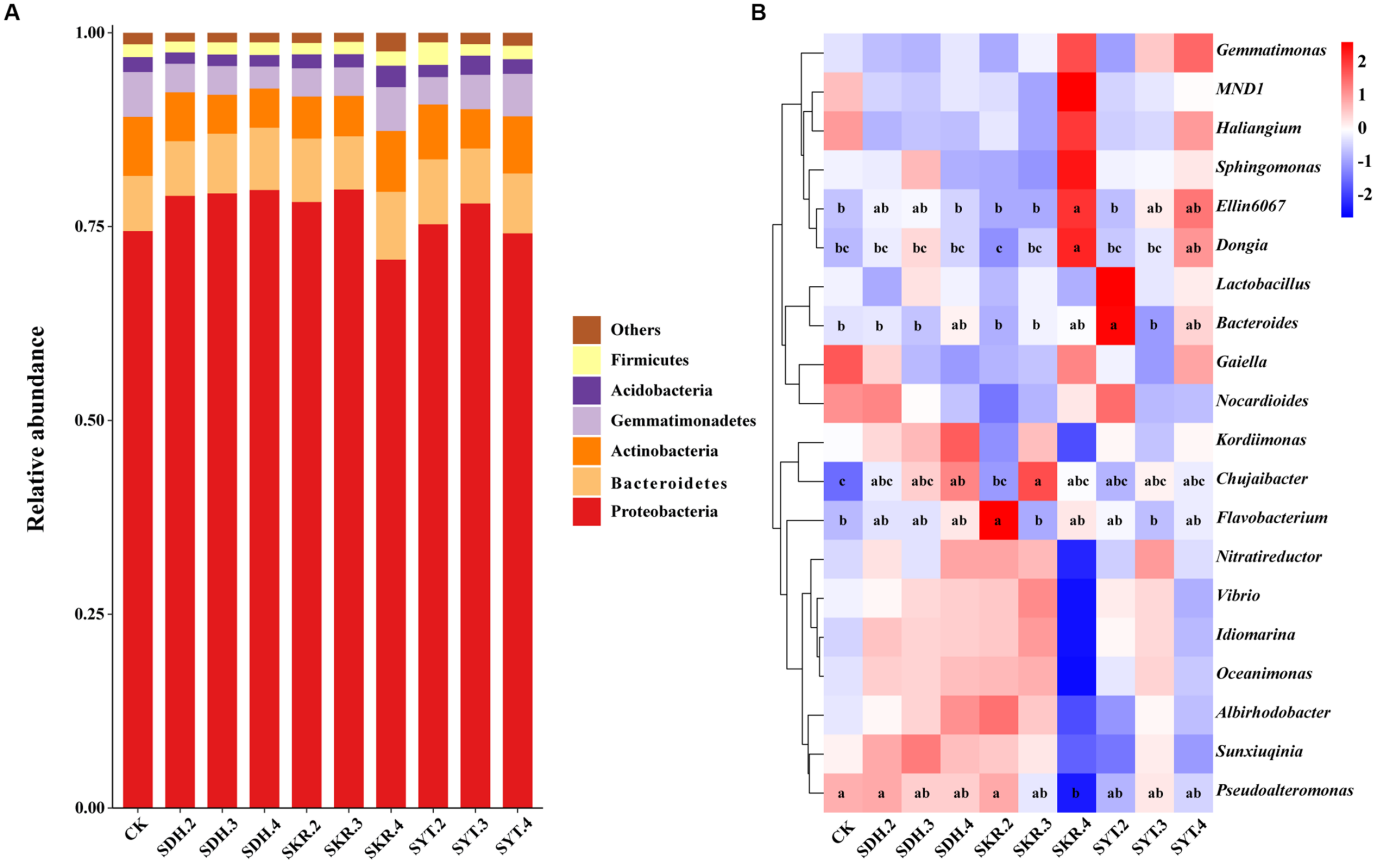
Effects of different fertilization treatments on the relative abundance of soil bacterial communities. The figure shows the top six phyla **(A)** and the top twenty genera of bacteria **(B)**. The phyla with relative abundances below 1% were grouped as “Others.” The definitions of CK, SDH.2, SDH.3, SDH.4, SKR.2, SKR.3, SKR.4, SYT.2, SYT.3, and SYT.4 are shown in Figure 1. The different letters in the figure indicate a significant difference at *p* < 0.05.

Among all samples, the top six bacterial phyla (relative abundance >1%) were Proteobacteria (76.8%), Bacteroidetes (7.7%), Actinobacteria (6.2%), Gemmatimonadetes (4.2%), Acidobacteria (1.9%), and Firmicutes (1.7%; Figure 2A). The relative abundance of Bacteroidetes in the high-level fertilization (SKR.4) was significantly (*p* < 0.05) higher than that of the medium-level fertilization (SKR.3). The relative abundance of Gemmatimonadetes in the SKR.4 treatment was significantly (*p* < 0.05) higher than that of the SDH.4 treatment. Compared to CK, the SKR.4 treatment significantly increased the relative abundance of Bacteroidetes, and the SYT.3 significantly increased the relative abundance of Acidobacteria (*p* < 0.05). However, the SDH.4 treatment significantly decreased the relative abundance of Gemmatimonadetes (*p* < 0.05; Supplementary Table S2).

At the genus level, the relative abundance of *Ellin6067* and *Dongia* in high-level fertilization (SKR.4) was significantly (*p* < 0.05) higher than that of the medium-level fertilization (SKR.3) and low-level fertilization (SKR.2). Moreover, SKR.4 treatment was led to a remarkable increase (1.0-fold increase) in the relative abundance of *Ellin6067* and a remarkable increase (2.0-fold increase) in the relative abundance of *Dongia* but a significant decrease in the abundance of *Pseudoalteromonas* relative to CK. Compared to CK, the SDH.4 and SKR.3 treatments significantly increased the relative abundance of *Chujaibacter* (*p* < 0.05). In addition, the relative abundance of *Bacteroides* in the SYT.2 treatment was significantly (*p* < 0.05) higher than that of the SDH.2 and SKR.2 treatments. Moreover, SKR.2 treatment significantly increased the relative abundance of *Flavobacterium*, and SYT.2 significantly increased the relative abundance of *Bacteroides* compared to CK (*p* < 0.05; Figure 2B).

**Figure 3:**
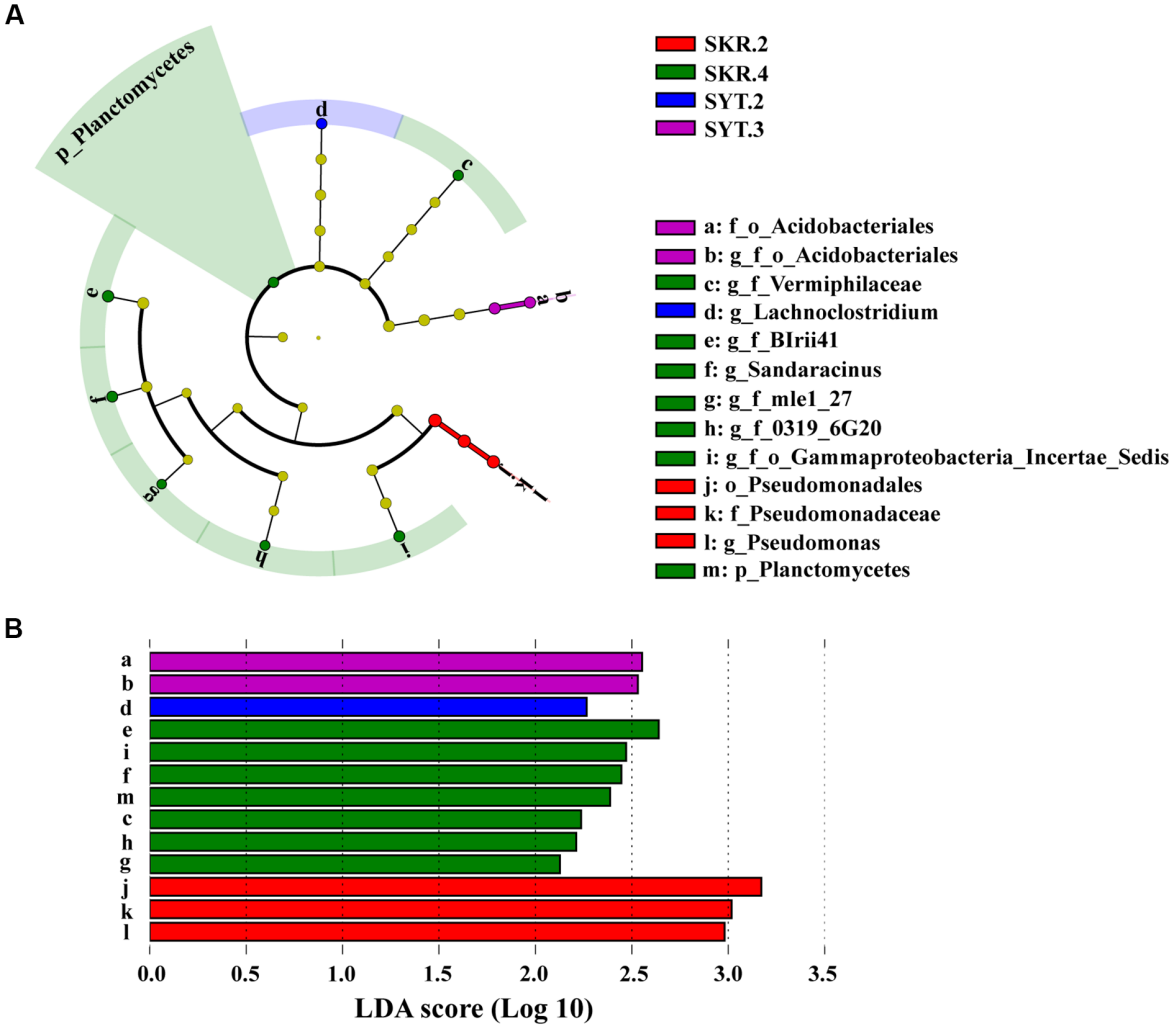
LEfSe analysis identified the taxa with significantly different abundances in different treatments (*p* < 0.05, LDA > 2.0). The figure demonstrates a cladogram of bacterial biomarkers **(A)** and a histogram of the LDA scores **(B)**. Circles of different colors indicate different treatments (red for SKR.2, green for SKR.4, blue for SYT.2, and purple for SYT.3). The circles from the inside to outside indicate bacteria from the kingdom to genus levels. The definitions of SKR.2, SKR.4, SYT.2, and SYT.3 are shown in Figure 1.

LEfSe analysis showed a total of 13 biomarkers of bacteria in the SKR.2, SKR.4, SYT.2, and SYT.3 treatments (Figure 3A). There were 3 bacterial taxa in the SKR.2 treatment, 7 bacterial taxa in the SKR.4 treatment, 1 bacterial taxon in the SYT.2 treatment, and 2 bacterial taxa in the SYT.3 treatment (Figure 3B). The number of bacterial taxa with significant differences in abundance in the SKR.4 treatment was the largest, which were g_f_Vermiphilaceae, g_f_BIrii41, g_f_mle1_27, g_f_0319_6G20, g_f_o_Gammaproteobacteria_Incertae_Sedis, *Sandaracinus* and Planctomycetes.

### 3.3. Relationship between soil properties, bacterial communities, and peach yield, and quality

**Figure 4:**
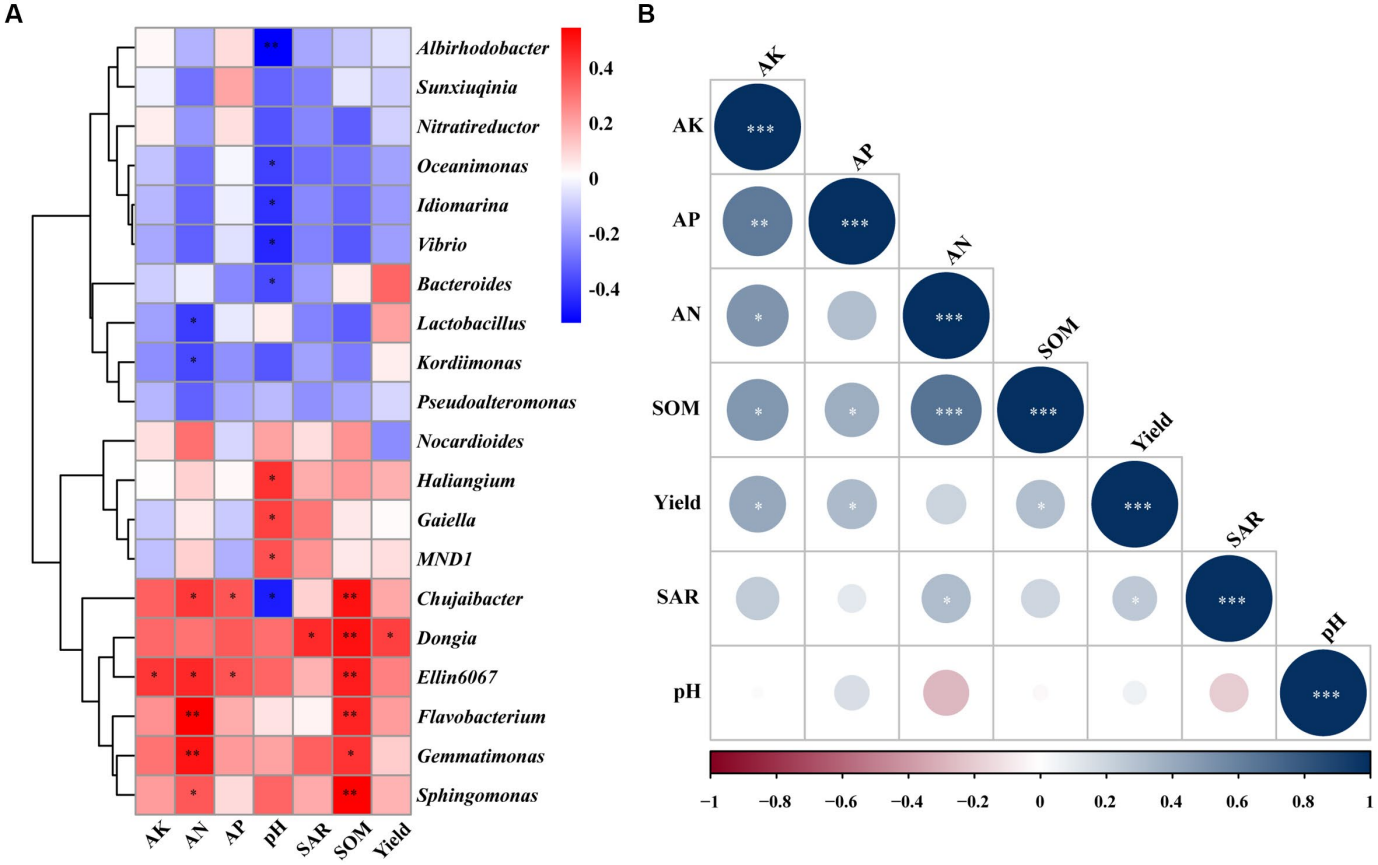
Correlation analysis of the top 20 genera of bacteria with chemical properties of soil and peach yield and quality **(A)**. Correlation analysis of the soil chemical properties and peach yield and quality **(B)**. AN, alkali-hydrolysable nitrogen; AK, available potassium; AP, available phosphorus; SOM, soil organic matter; and SAR, sugar/acid ratio. The correlation analysis used the Spearman correlation coefficient. * significance at *p* < 0.05, ** significance at *p* < 0.01, *** significance at *p* < 0.001.

The correlation analysis showed that there was a significant correlation between different bacterial genera and soil chemical properties (Figure 4A). SOM (*p* < 0.01), AK, AN, and AP (*p* < 0.05) were significantly positively correlated with *Ellin6067*. There is a significant positive correlation between SOM (*p* < 0.01), AN, and AP with *Chujaibacter*, while pH exhibited a significant negative correlation with *Chujaibacter* (*p* < 0.05). Moreover, SOM was significantly positively correlated with *Sphingomonas*, *Flavobacterium*, *Dongia* (*p* < 0.01), and *Gemmatimonas* (*p* < 0.05). In contrast, AN and pH were significantly negatively correlated with *Kordiimonas*, *Lactobacillus, Bacteroides*, *Vibrio*, *Idiomarina*, *Oceanimonas* (*p* < 0.05), and *Albirhodobacter* (*p* < 0.01). In addition, *Dongia* presented a significant positive correlation with yield and SAR (*p* < 0.05). AK, AP, and SOM were significantly positively correlated with yield (*p* < 0.05). Moreover, SAR was significantly positively correlated with AN (*p* < 0.05; Figure 4B).

### 3.4. Network analysis of soil bacterial communities

**Figure 5:**
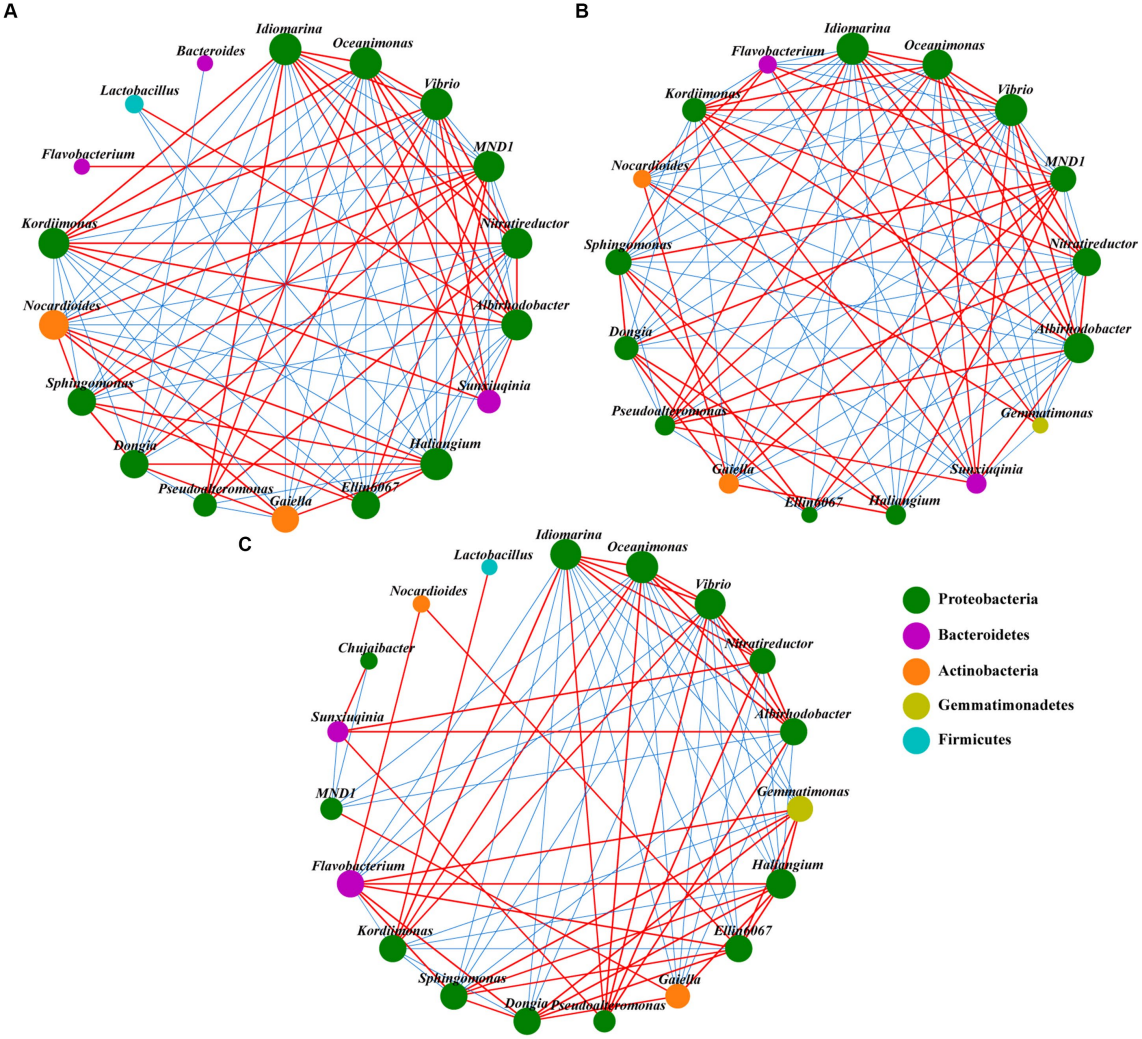
Network analysis of soil bacterial communities (top 20 genera) under SDH **(A)**, SKR **(B)**, and SYT **(C)** treatments. The red line between nodes indicates a positive correlation, and the blue line between nodes indicates a negative correlation. The size of the nodes represents the degree of a genus of bacteria, and they are colored based on their classification at the phylum level. SDH, application of Dahua organic fertilizer; SKR, application of Kerui organic fertilizer; and SYT, application of Yite commercial organic fertilizer.

The SDH network had 18 nodes and 91 edges (Figure 5A). The SKR network had the least nodes and the most edges, with 17 and 100, respectively (Figure 5B). However, the SYT network has the most nodes and the least edges, with 19 and 80, respectively (Figure 5C). In addition, the ratio of negative links was higher than the ratio of positive links in the SDH and SKR networks and the opposite for the SYT networks. Moreover, the ratio of positive links was highest in the SYT network, while the ratio of negative links was higher in the SKR network (Supplementary Table S3). The number of positive links and negative links of *MND1* in the SDH and SKR networks was higher than that in the SYT network. Compared to the SYT network, the number of positive links decreased and the number of negative links increased of *Dongia* in SDH and SKR networks. The number of positive links and negative links of *Gemmatimonas* and *Flavobacterium* in the SDH network were the lowest among the three networks (Supplementary Table S4).

### 3.5. Functional prediction of soil bacterial communities

**Figure 6:**
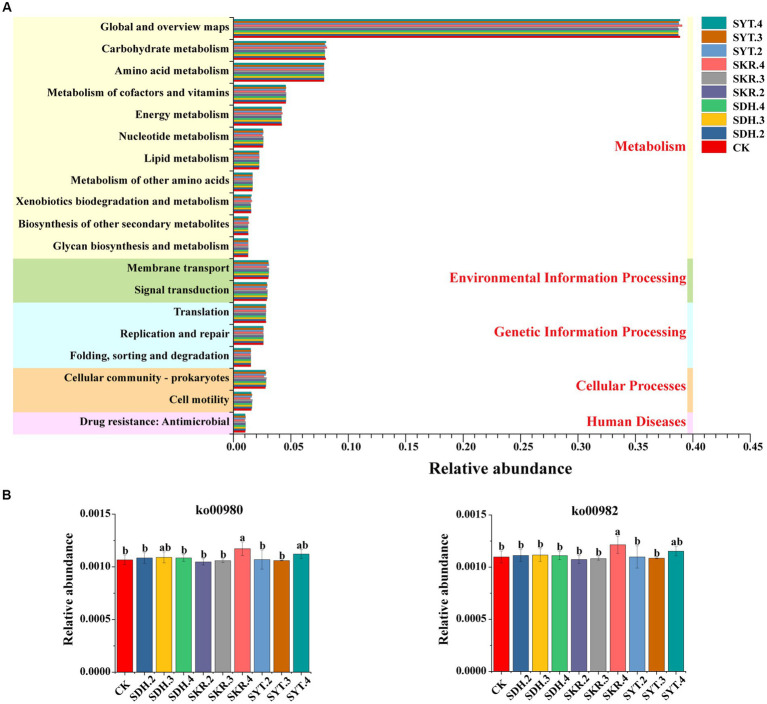
Bacterial KEGG pathway prediction by PICRUSt2. The figure demonstrates the level 2 function categories **(A)** and the relative abundance of ko00980 and ko00982 under different treatments **(B)**. The definitions of CK, SDH.2, SDH.3, SDH.4, SKR.2, SKR.3, SKR.4, SYT.2, SYT.3, and SYT.4 are shown in Figure 1. The different letters in the figure indicate a significant difference at *p* < 0.05. ko00980: Metabolism of xenobiotics by cytochrome P450; ko00982: Drug metabolism – cytochrome P450.

The prediction results showed that 19 pathways (level 2, with a relative abundance of >1%) were identified, including 11 pathways of metabolism (level 1), two pathways of environmental information processing (level 1), three pathways of genetic information processing (level 1), two pathways of cellular processes (level 1) and one pathway of human diseases (level 1; Figure 6A). Although there were no significant differences in the 19 pathways under the 9 fertilization treatments compared to CK, the relative abundance of xenobiotic biodegradation and metabolism increased by 5.01% under the SKR.4 treatment. Furthermore, the relative abundance of xenobiotic biodegradation and metabolism under high-level fertilization (SKR.4) was significantly (*p* < 0.05) higher than that under medium-level fertilization (SKR.3). However, there were no significant differences when comparing SKR.4 with SDH.4 and SYT.4 treatments. The level 3 KEGG functional categories of xenobiotic biodegradation and metabolism were further analyzed, and the relative abundances of ko00980 and ko00982 were significantly higher under SKR.4 treatment than under CK (*p* < 0.05; Figure 6B). Additionally, the relative abundances of ko00980 and ko00982 under high-level fertilization (SKR.4) were significantly (*p* < 0.05) higher than that in the medium-level fertilization (SKR.3) and the low-level fertilization (SKR.2), and significantly (*p* < 0.05) higher than that in the SDH.4 treatment, but no significant difference compared to the SYT.4 treatment.

Interestingly, in the lower relative abundance of the level 2 pathway, the relative abundance of aging under the SKR.4 treatment is significantly higher (*p* < 0.05) than that of the other nine treatments. Further analysis of the level 3 KEGG functional categories of aging revealed similar findings, indicating that the relative abundance of ko04212 in the SKR.4 treatment was significantly (*p* < 0.05) higher than the other nine treatments (Supplementary Figure S1).

**Figure 7:**
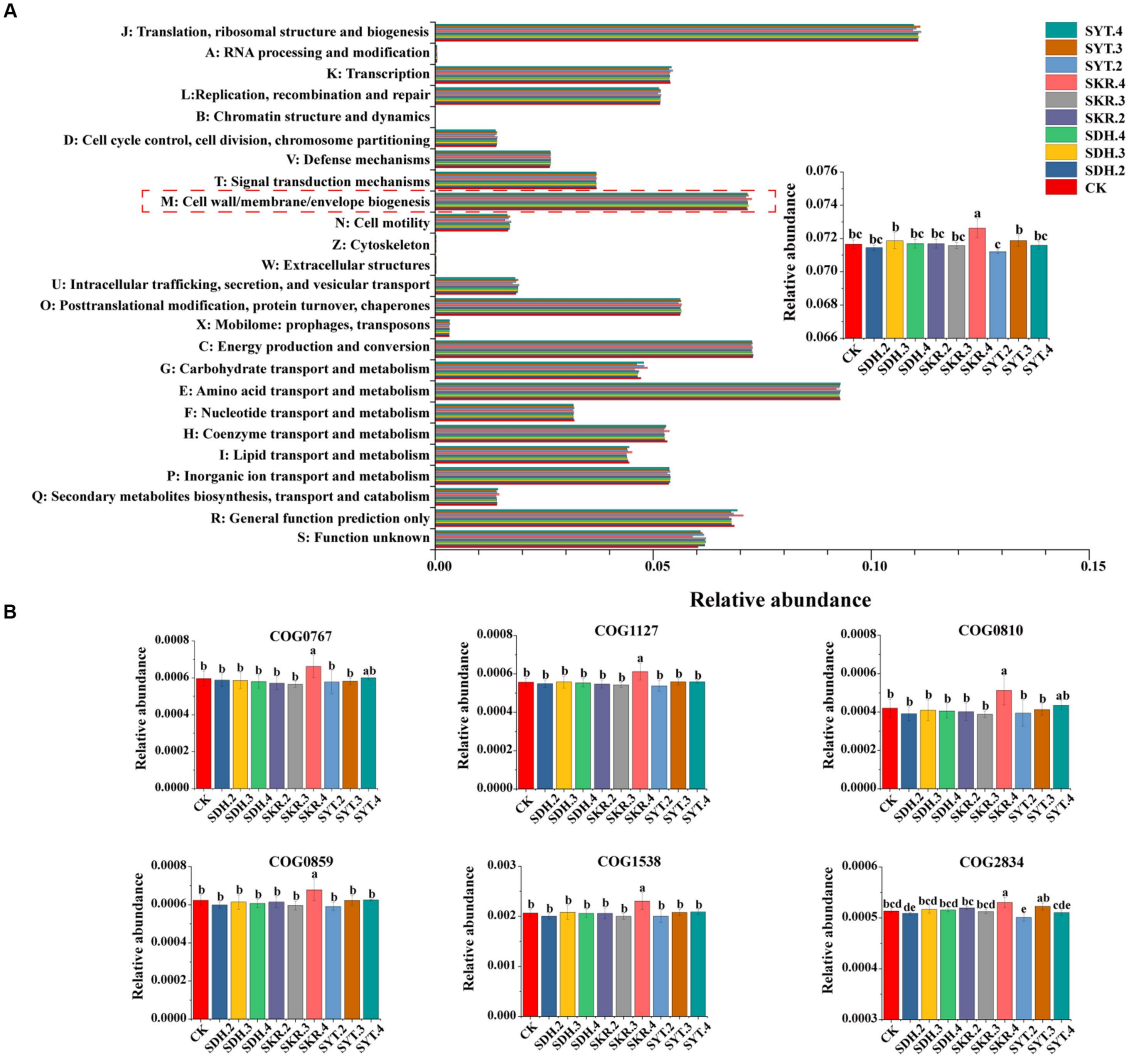
Bacterial COG functional category prediction by PICRUSt2. The figure demonstrates the COG functional categories **(A)** and the relative abundance of COG0767, COG127, COG0810, COG0859, COG1538, and COG2834 under different treatments **(B)**. The definitions of CK, SDH.2, SDH.3, SDH.4, SKR.2, SKR.3, SKR.4, SYT.2, SYT.3, and SYT.4 are shown in Figure 1. The different letters in the figure indicate a significant difference at *p* < 0.05. COG0767: ABC-type transporter Mla maintaining outer membrane lipid asymmetry, permease component MlaE; COG1127: ABC-type transporter Mla maintaining outer membrane lipid asymmetry, ATPase component MlaF; COG0810: Periplasmic protein TonB, links inner and outer membranes; COG0859: ADP-heptose:LPS heptosyltransferase; COG1538: Outer membrane protein TolC; COG2834: outer membrane lipoprotein-sorting protein.

The prediction results showed that 25 categories were identified. The relative abundance of cell wall/membrane/envelope biogenesis in the SKR.4 treatment was significantly (*p* < 0.05) higher than CK, as well as the medium-level fertilization (SKR.3) and low-level fertilization (SKR.2). Moreover, it was significantly (*p* < 0.05) higher than the other two organic fertilizers (SDH.4 and SYT.4; Figure 7A). Further analysis found that SKR.4 treatment significantly (*p* < 0.05) increased the relative abundance of COG0767, COG1127, COG0810, COG0859, COG1538, and COG2834 in cell wall/membrane/envelope biogenesis compared to CK, the medium-level fertilization (SKR.3) and low-level fertilization (SKR.2; Figure 7B).

## 4. Discussion

The results of this study showed that soil chemical properties changed significantly after the application of organic fertilizer. Specifically, the application of organic fertilizer (SDH, SKR, and SYT) significantly increased soil AN, AP, AK, and SOM compared to CK, which is consistent with findings from previous studies (Baldi et al., 2016; Kobierski et al., 2017; Su et al., 2021). This indicates that PBOF can enhance the nutrient availability as well as the fertility of the soil, which benefits the growth and development of plants. The increasing application levels of three types of organic fertilizers did not have a significant impact on AN, AP, AK, and SOM, except for AK in SYT.3 and SYT.4 treatments which were significantly higher than that of SYT.2 treatment, which may be related to the fact that organic fertilizers were applied for only 1 year (Table 2). In a two-year research study, there were significant differences between different organic fertilization levels in soil nutrients (Chen et al., 2022). Interestingly, all fertilization treatments did not have a significant effect on soil pH compared to CK, which disagreed with previous findings (Tang et al., 2020). This discrepancy might be due to differences in the soil type (Wei et al., 2017). By comparing the three organic fertilizers, it was found that the two PBOF had similar effects to commercial organic fertilizers in affecting soil chemical properties and improving soil nutrients.

In addition, the peach yield increased significantly after the application of organic fertilizer (Table 1). Similar results can be found in apple (Milošević et al., 2022), pear (Wang Z. H. et al., 2022), pear-jujube (Ye S. L. et al., 2022), red pitaya (Chen et al., 2022), and kiwifruit (Liu et al., 2020). In our study, the application of organic fertilizer also significantly increased the SSC/TA ratio of peach fruit (Table 1). The eating quality of peaches is mainly related to the sugar/acid ratio. Peaches with high sugar/acid ratio can potentially achieve higher retail values than peaches with low sugar/acid ratio (Minas et al., 2018). Therefore, the application of organic fertilizer increased the yield of peach fruit and improve the quality of peach fruit, which was beneficial to increase the economic income of farmers. Furthermore, with the increase in fertilization levels, the peach fruit yield and sugar/acid ratio also gradually increased, which might be attributed to the improvement of soil nutrients. Similar to commercial organic fertilizer, PBOF can also improve the yield and quality of peach fruit. Importantly, according to government policy, fruit farmers can exchange organic fertilizer through peach branches, which is estimated to be 1.5 tons of organic fertilizer per ton of peach branches, which brings great economic value to fruit farmers and reduces costs.

We further analyzed changes in soil bacterial communities. The application of organic fertilizer (SDH, SKR, and SYT) did not have a significant effect on soil bacterial community diversity compared to CK (Table 3). The Chao1 and Shannon indices also did not change significantly after the application of organic fertilizer in other studies (Jiao et al., 2021; Tan et al., 2023). However, another study showed that organic fertilizer significantly decreased the Chao1 and Shannon indices (Wang T. et al., 2022). Research by Wang et al. (2017) showed that organic fertilizer significantly increased the Chao1 index but had no significant effect on the Shannon index. This may be due to differences in soil and organic fertilizer types. With the exception of SKR.4 and SYT.4 treatments, all fertilization treatments resulted in a decrease in Chao1 and Shannon indices (Table 3). Moreover, the reduction effect of PBOF was more obvious, which might imply a negative impact of PBOF on soil microbial community diversity.

Unlike the results of soil bacterial community diversity, the composition of soil bacterial communities changed significantly after the application of organic fertilizer. At the phylum level, Proteobacteria, Bacteroidetes, Actinobacteria, Gemmatimonadetes, Acidobacteria, and Firmicutes were the dominant bacteria, which were similar to those found in other studies after the application of organic fertilizer (Joa et al., 2014; Chen et al., 2020; Jiao et al., 2021; Wang T. et al., 2022). However, the relative abundance of certain phyla varied among different treatments. For instance, the SKR.4 and SYT.3 treatments significantly increased the relative abundance of Bacteroidetes and Acidobacteria, respectively, but the SDH.4 treatment significantly decreased the relative abundance of Gemmatimonadetes (Supplementary Table S2). At the genus level, SKR.2 treatment significantly increased the relative abundance of *Flavobacterium*, which is reported to often harbor amylase, cellulase, and chitinase, which are capable of degrading a wide range of organic compounds (Kolton et al., 2016). Furthermore, SDH.4 and SKR.3 treatments significantly increased the relative abundance of *Chujaibacter* (Figure 2B). *Chujaibacter* is closely related to heavy metal metabolism (Liang et al., 2021). In another study, Zn application impacted the relative abundance of *Ellin6067*, a nitrosifying bacterium that converts NH_4_^+^ to NO_3_^−^, providing an accessible nitrogen source for plants (Lv et al., 2022). Additionally, *Dongia* has been reported to be responsible for suppressing soil-borne pathogens (Han et al., 2019). In our study, SKR.4 treatment significantly increased the relative abundance of *Ellin6067* and *Dongia* (Figure 2B). Further analyzing the results of LEfSe, we found that *Sandaracinus* was a biomarker in the SKR.4 treatment (Figure 3). *Sandaracinus* is a myxobacteria, and a previous study found that the application of organic fertilizer significantly increased the content of soil organic matter, which may promote the growth of myxobacteria (Wang et al., 2020). Interestingly, the soil organic matter content of the SKR.4 treatment was the highest of all the treatments (Table 2). Moreover, *Pseudomonas* was a biomarker in the SKR.2 treatment (Figure 3). *Pseudomonas* can restrain the growth of pathogens and degrade pollutants, thereby favoring plant growth (Liu et al., 2020).

These findings highlighted the distinct impacts of different organic fertilizers and fertilization levels on the community composition and abundance of soil bacteria, which might be due to differences in soil chemical properties and the introduction of exogenous microorganisms from organic fertilizers. For the latter, it has been shown that exogenous microbes introduced to the soil from organic fertilizers affect the soil microbial communities. For example, some fungi introduced to the soil from manure were closely related to pathogen-antagonists (Sun et al., 2016). In conclusion, these results indicated that the application of organic fertilizer significantly increased the relative abundance of beneficial bacteria in the soil, which had potential functions in organic compound degradation, heavy metal metabolism, nitrogen supply, and pathogen suppression, thereby promoting soil health and plant growth and revealed that the application of organic fertilizers, including SDH, SKR, and SYT, at different fertilization levels, resulted in significant changes in soil bacterial community.

Many studies have shown a strong correlation between soil chemical properties, soil bacterial communities, and fruit yield and quality (Liu et al., 2020; Wang Z. H. et al., 2022; Zhang et al., 2022b). The same results were found in our correlation analysis, in which many soil bacteria were significantly positively correlated with soil chemical properties and peach yield and quality (Figure 4A). In addition, peach yield and quality were also significantly positively correlated with soil chemical properties (Figure 4B). Nitrogen(N) has a great impact on fruit quality and fruit tree productivity. If N is deficient, the yield will be reduced and the quality of fruits will be poor (Minas et al., 2018). This is consistent with our study that AN was positively correlated with yield and sugar/acid ratio. Moreover, the correlation analysis revealed a positive relationship between AK and both yield and sugar/acid ratio, which same as previous studies showing the beneficial effects of suitable potassium fertilization on internal fruit quality. It can be attributed to the enhancement of photosynthesis rates and the translocation of soluble sugars and organic acids (Minas et al., 2018). These results indicated that the application of organic fertilizers (SDH, SKR, and SYT) promoted peach fruit quality and yield, most likely due to changed soil chemical properties, enhanced soil nutrients, and affected soil bacterial community composition.

The network analysis showed that the response of networks to different organic fertilizers was different for bacterial communities (Figure 5; Supplementary Table S3). Similar results were found in previous studies (Wang et al., 2017; Jin et al., 2022). The decrease in the number of network edges may be due to the increased nutrient supply caused by the application of organic fertilizer, which reduces the difficulty of microorganisms to obtain nutrients and thus the association between microorganisms. In addition, the networks of the two PBOF had a higher ratio of negative links than the network of commercial organic fertilizer. This may be because the introduction of exogenous microorganisms in PBOF has affected the local microbial community (Sun et al., 2020). Moreover, the higher ratio of negative links may be caused by competitive or antagonistic interactions between soil bacteria. This may reduce the invasion of pathogenic microorganisms in the soil and contribute to maintaining the health of the soil ecosystem, and as reported by Li et al. (2019), antagonistic interactions between microorganisms may suppress pathogen invasions. Furthermore, the Kerui PBOF increased the number of positive and negative links of the beneficial bacteria *Gemmatimonas* and *Flavobacterium*, compared to the Dahua PBOF, and the network density of the SKR network was the highest among the three networks (Supplementary Tables S3, S4). Overall, the Kerui PBOF may enhance microbial co-occurrence pattern.

The soil bacterial community functions of different treatments were predicted by PICRUSt2. The KEGG results showed that SKR.4 treatment enhanced xenobiotic biodegradation and metabolism and aging (Figure 6B; Supplementary Figure S1). Zhang et al. (2022a) also found the same result after applying organic fertilizer. Further analysis of the level 3 pathways of xenobiotic biodegradation and metabolism and aging revealed that SKR.4 treatment enhanced the metabolism of xenobiotics by cytochrome P450, drug metabolism – cytochrome P450, and longevity regulating pathway – worm (Figure 6B; Supplementary Figure S1). This may indicate that the high-level application of Kerui PBOF influences the degradation and metabolism of exogenous compounds, such as environmental pollutants, through the involvement of cytochrome P450 enzymes. Moreover, the high-level application of Kerui PBOF has the potential to influence the aging of nematodes, which in turn affects bacterial communities and promotes plant growth. In addition, the COG results showed that SKR.4 treatment enhanced cell wall/membrane/envelope biogenesis (Figure 7A). Similar results were found in the study of Ma et al. (2020) and higher available potassium and organic matter content promoted cell wall/membrane/envelope biogenesis. In our study among all fertilization treatments, SKR.4 treatment had the highest SOM and higher AK (Table 2). Further analysis showed that the relative abundance of six COGs in cell wall/membrane/envelope biogenesis increased, suggesting that the high-level application of Kerui PBOF may enhance the composition of the outer membrane of bacteria, which in turn promoted the growth of bacteria (Figure 7B). Overall, these results showed that the application of organic fertilizer affected bacterial community function, especially under the SKR.4 treatment. However, further research is required to fully understand the underlying mechanisms and broader implications of these findings.

## 5. Conclusion

This study showed that PBOF could effectively improve the yield and quality of peach fruit and increase soil nutrients. It also changed the soil bacterial community composition and promoted the growth of beneficial bacteria. Moreover, the application of PBOF enhanced the soil bacterial co-occurrence pattern and the potential function of bacterial communities to degrade exogenous compounds. In general, PBOF could achieve similar results to the application effect of commercial organic fertilizer, and the SKR.4 treatment (Kerui PBOF, 5,120 kg/667 m^2^) was the better choice for the peach orchard in this study. These results will help establish scientific fertilization strategies in peach orchards, ensuring the yield and quality of peach fruit while promoting the sustainable production of peaches. In addition, thanks to the local policy of encouraging the use of PBOF, the use cost of PBOF is lower than commercial organic fertilizer, which is conducive to the development of ecological agriculture.

## Data availability statement

The data presented in the study are deposited in the NCBI repository, accession number PRJNA937092.

## Author contributions

CL and DH developed the concept of this study and are main contributors to writing the manuscript. HY was responsible for performing the field experiments. DH was responsible for performing the lab experiments. CL and ZL performed the data analysis and prepared the figures. CG and YL contributed to the manuscript edit and review. All authors contributed to the article and approved the submitted version.

## Funding

This study was supported by the Deloitte Charity Foundation – Demonstration of Low-Carbon Circular Agriculture in Pinggu District (202101226).

## Conflict of interest

The authors declare that the research was conducted in the absence of any commercial or financial relationships that could be construed as a potential conflict of interest.

## Publisher’s note

All claims expressed in this article are solely those of the authors and do not necessarily represent those of their affiliated organizations, or those of the publisher, the editors and the reviewers. Any product that may be evaluated in this article, or claim that may be made by its manufacturer, is not guaranteed or endorsed by the publisher.

## Supplementary material

The Supplementary material for this article can be found online at: https://www.frontiersin.org/articles/10.3389/fmicb.2023.1223420/full#supplementary-material

## Footnotes

^1^http://www.cloudtutu.com

^2^https://cloud.oebiotech.com

^3^https://www.bioincloud.tech
