# Supplementary material for: Effects of peach branch organic fertilizer on the soil microbial community in peach orachards
Source: Front Microbiol. 2023 Jul 7;14:1223420. doi: 10.3389/fmicb.2023.1223420 (PMC10361838; doi:10.3389/fmicb.2023.1223420)
Supplement: Supplementary file 1 [file Data_Sheet_1.docx]

Supplementary Material

Effects of peach branch organic fertilizer on the soil microbial community in peach orachards

Chenyu Liu, Defeng Han, Haiqing Yang, Zhiling Liu, Chengda Gao^*^, Yueping Liu^*^

*** Correspondence:** Chengda Gao: 1364072545@qq.com, Yueping Liu: liuyueping@bua.edu.cn

# Supplementary Figures and Tables

## Supplementary Figures


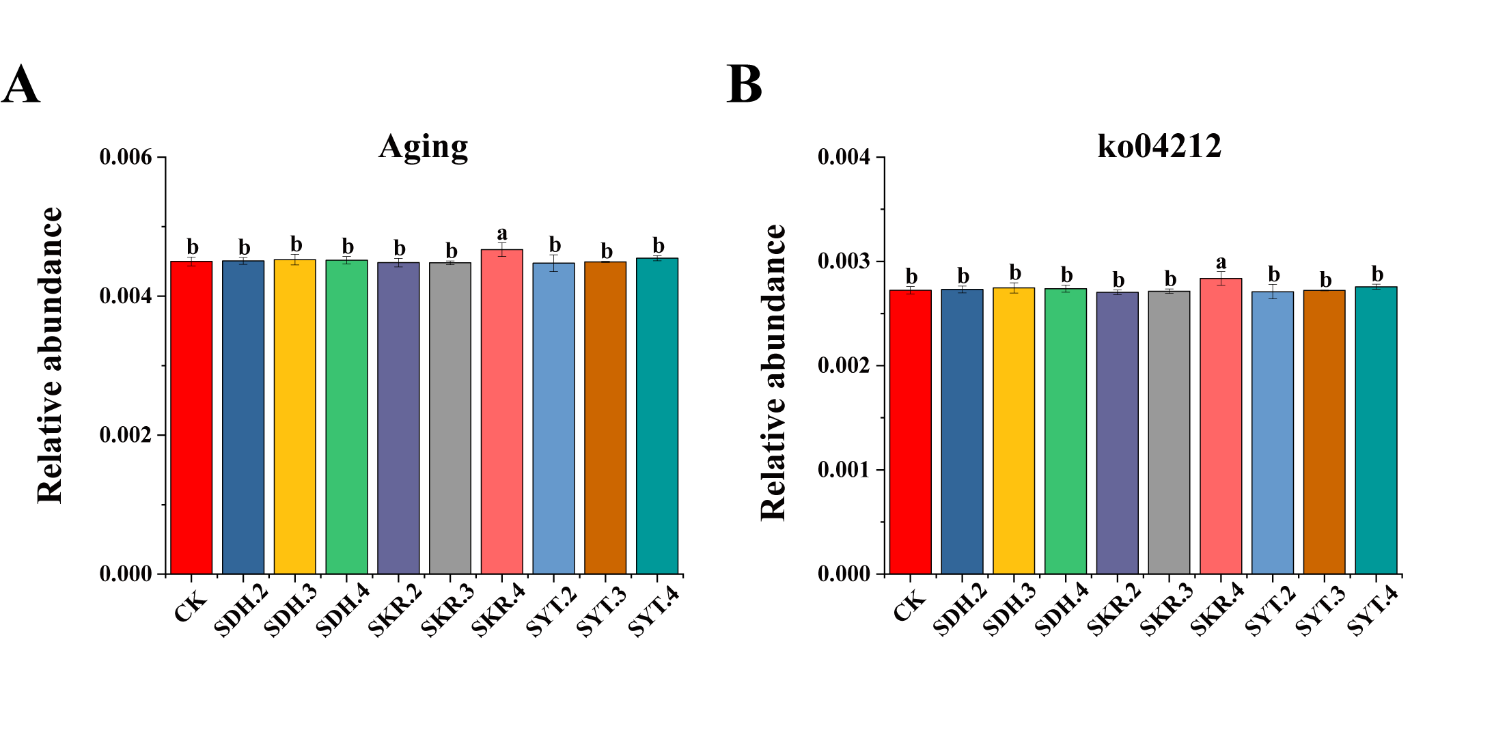


**Supplementary Figure 1.** Differences in relative abundance of Aging and ko04212 under different fertilization treatments. ko04212: Longevity regulating pathway - worm.

## Supplementary Tables

**Supplementary Table 1.** Chemical properties of soil and three organic fertilizers before the experiment

| **Chemical properties** | **Soil** | **Dahua** | **Kerui** | **Yite** |
| --- | --- | --- | --- | --- |
| SOM (g/kg) | 19.79 | 212.65 | 444.20 | 283.96 |
| AN (mg/kg) | 126.70 | 840.56 | 1327.67 | 1099.54 |
| AP (mg/kg) | 145.98 | 193.98 | 353.84 | 644.27 |
| AK (mg/kg) | 399.07 | 11606.52 | 8954.88 | 20781.60 |
| pH | 7.37 | - | - | - |
| Ca (g/kg) | 3.47 | 12.63 | 30.76 | 14.16 |
| Cu (mg/kg) | 18.49 | 36.06 | 68.68 | 60.34 |
| Fe (g/kg) | 30.01 | 19.39 | 13.27 | 14.66 |
| Mn (g/kg) | 0.52 | 0.52 | 0.44 | 0.48 |
| Mg (g/kg) | 0.86 | 3.62 | 5.64 | 5.59 |
| Zn (mg/kg) | 93.38 | 275.09 | 298.53 | 459.97 |

**Supplementary Table 2.** Analysis of differences in relative abundance of the top six phyla of bacteria

| **Treatment** | **Proteobacteria** | **Bacteroidetes** | **Actinobacteria** | **Gemmatimonadetes** | **Acidobacteria** | **Firmicutes** |
| --- | --- | --- | --- | --- | --- | --- |
| CK | 0.744 ± 0.065a | 0.071 ± 0.005b | 0.076 ± 0.036a | 0.058 ± 0.025a | 0.019 ± 0.008bc | 0.017 ± 0.004ab |
| SDH.2 | 0.790 ± 0.037a | 0.071 ± 0.003b | 0.063 ± 0.019a | 0.037 ± 0.012ab | 0.015 ± 0.005c | 0.014 ± 0.002b |
| SDH.3 | 0.793 ± 0.041a | 0.077 ± 0.009ab | 0.050 ± 0.018a | 0.037 ± 0.009ab | 0.015 ± 0.005c | 0.016 ± 0.001ab |
| SDH.4 | 0.797 ± 0.034a | 0.080 ± 0.002ab | 0.050 ± 0.016a | 0.029 ± 0.009b | 0.015 ± 0.005c | 0.016 ± 0.004ab |
| SKR.2 | 0.782 ± 0.060a | 0.082 ± 0.016ab | 0.054 ± 0.021a | 0.037 ± 0.012ab | 0.018 ± 0.003bc | 0.015 ± 0.003b |
| SKR.3 | 0.798 ± 0.033a | 0.069 ± 0.004b | 0.052 ± 0.011a | 0.037 ± 0.007ab | 0.017 ± 0.007bc | 0.016 ± 0.002ab |
| SKR.4 | 0.707 ± 0.061a | 0.087 ± 0.003a | 0.079 ± 0.029a | 0.056 ± 0.016a | 0.028 ± 0.012b | 0.018 ± 0.003ab |
| SYT.2 | 0.753 ± 0.092a | 0.084 ± 0.019ab | 0.071 ± 0.047a | 0.035 ± 0.028ab | 0.016 ± 0.006bc | 0.029 ± 0.023a |
| SYT.3 | 0.780 ± 0.041a | 0.071 ± 0.002b | 0.051 ± 0.010a | 0.044 ± 0.017ab | 0.051 ± 0.010a | 0.015 ± 0.001b |
| SYT.4 | 0.741 ± 0.046a | 0.077 ± 0.009ab | 0.074 ± 0.014a | 0.055 ± 0.016ab | 0.019 ± 0.004bc | 0.017 ± 0.004ab |

**Supplementary Table 3.** Network topological properties of soil bacterial communities in peach orchards under different fertilization treatments

| **Network topological properties** | **SDH** | **SKR** | **SYT** |
| --- | --- | --- | --- |
| Nodes | 18 | 17 | 19 |
| Edges | 91 | 100 | 80 |
| Average degree | 10.11 | 11.76 | 8.42 |
| Network density | 0.59 | 0.74 | 0.47 |
| Positive links (%) | 46.15 | 44.00 | 53.75 |
| Negative links (%) | 53.85 | 56.00 | 46.25 |

**Supplementary Table 4.** The number of positive and negative links of *MND1*, *Dongia*, *Gemmatimonas* and *Flavobacterium* under different fertilization treatments

| **Treatment** | ***MND1*** | | ***Dongia*** | | ***Gemmatimonas*** | | ***Flavobacterium*** | |
| --- | --- | --- | --- | --- | --- | --- | --- | --- |
|  | **Positive links** | **Negative links** | **Positive links** | **Negative links** | **Positive links** | **Negative links** | **Positive links** | **Negative links** |
| SDH | 6 | 7 | 4 | 7 | 0 | 0 | 1 | 0 |
| SKR | 6 | 7 | 5 | 7 | 2 | 6 | 4 | 5 |
| SYT | 1 | 5 | 6 | 4 | 5 | 4 | 6 | 4 |
